# Selective suppression and recall of long-term memories in *Drosophila*

**DOI:** 10.1371/journal.pbio.3000400

**Published:** 2019-08-27

**Authors:** Dominique Siegenthaler, Benjamin Escribano, Vanessa Bräuler, Jan Pielage

**Affiliations:** Division of Neurobiology and Zoology, University of Kaiserslautern, Kaiserslautern, Germany; Stony Brook University, UNITED STATES

## Abstract

Adaptive decision-making depends on the formation of novel memories. In *Drosophila*, the mushroom body (MB) is the site of associative olfactory long-term memory (LTM) storage. However, due to the sparse and stochastic representation of olfactory information in Kenyon cells (KCs), genetic access to individual LTMs remains elusive. Here, we develop a cAMP response element (CRE)-activity–dependent memory engram label (CAMEL) tool that genetically tags KCs responding to the conditioned stimulus (CS). CAMEL activity depends on protein-synthesis–dependent aversive LTM conditioning and reflects the time course of CRE binding protein 2 (CREB2) activity during natural memory formation. We demonstrate that inhibition of LTM-induced CAMEL neurons reduces memory expression and that artificial optogenetic reactivation is sufficient to evoke aversive behavior phenocopying memory recall. Together, our data are consistent with CAMEL neurons marking a subset of engram KCs encoding individual memories. This study provides new insights into memory circuitry organization and an entry point towards cellular and molecular understanding of LTM storage.

## Abstract 2

A novel genetic approach enables the visualization and manipulation of memory engram cells in Drosophila, providing a key methodological opportunity to characterize associative memory at the cellular and circuit level.

## Introduction

Successful adaptation to aversive stimuli through the formation of associative memories is essential for the survival of most animals. The physical correlate of these memories, the memory engram, represents long-lasting alterations of neurons and circuits that enable precise recall of stored information to modulate behavior [1–3]. *Drosophila* aversive olfactory conditioning has been successfully used in the past to unravel the molecular mechanisms underlying long-term associative memory [4–7].

The mushroom body (MB), the site of associative olfactory learning in insects, consists of 7 types (αβc, αβs, αβp, α’β’m, α’β’ap, γm, and γd) of Kenyon cells (KCs) that project into specific layers in the α/β, α’/β’, and γ MB lobes that represent functional domains dedicated to different aspects of memory acquisition, storage, and retrieval [8–19].

Olfactory information from approximately 50 antennal lobe glomeruli is provided in a stochastic manner via projection neurons (PNs) to approximately 2,000 KCs [20–24]. KCs then transmit the information to only 34 MB output neurons (MBONs) in 15 anatomically defined lobular compartments [18,19]. Consistent with this anatomical architecture, MBONs are broadly tuned to odors, whereas olfactory sensory representation in KCs is sparse and thus well suited for memory association [25–28]. The KC > MBON synaptic compartments are selectively innervated by dopaminergic neurons (DANs) that provide reinforcement signals of positive or negative valence during associative memory formation [18,29–32]. Temporal pairing of an odor with an aversive stimulus results in KC activity that coincides with dopamine release and induces synaptic depression of the KC > MBON synapse in a compartment-specific manner [33–35]. Because MBONs are known to direct approach or avoidance behavior [36,37], KC > MBON plasticity is thought to be sufficient to modulate behavior and store olfactory memories [38,39]. The recent complete synaptic reconstruction of the adult MB α lobe [40] and of the entire adult brain [41] in combination with genetic access to MBONs and DANs [18] provide a unique framework to unravel the cellular and circuit mechanisms underlying long-term memory (LTM).

Prior studies demonstrated that one subtype of KCs, the αβ surface neurons (500 cells), is essential for LTM recall [9], indicating that olfactory LTMs are stored within these neurons. The stochastic PN > KC synaptic connectivity and the sparse representation of olfactory information within a genetically identical subpopulation of KCs currently precludes the identification and characterization of the KCs encoding individual LTMs. In mice, activity-dependent techniques based on the transcriptional activity of c-Fos enabled successful tagging and manipulation of engram cells [42–44], and a similar approach has recently been described in Drosophila [45]. In addition to c-Fos, cellular consolidation of LTM requires protein synthesis and activity of the cAMP response element (CRE)-binding protein CREB in mice, *Aplysia* and *Drosophila* MBs [5,45–56]. In this study, we generate a novel engram-tagging tool based on CRE-dependent transcriptional activity to specifically manipulate KCs encoding individual LTMs.

## Results

**Fig 1:**
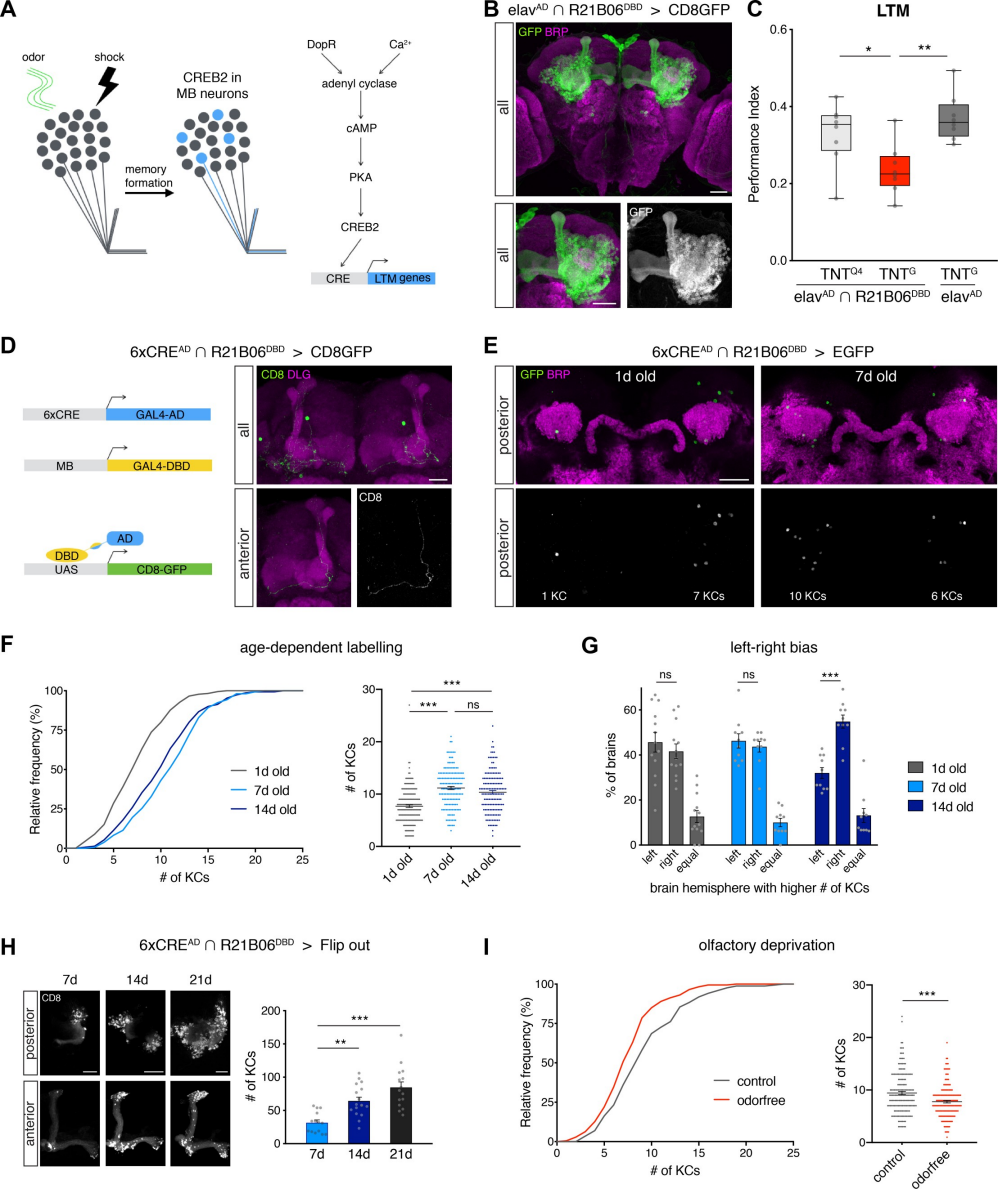
Experience-dependent tagging of KCs. (A) Schematic of the CAMEL-tool principle enabling CREB2-dependent tagging of putative memory engram neurons (see also panel D). (B) The R21B06^DBD^ line specifically labels αβ surface and γ neurons when combined with the pan-neuronal elav^AD^ line. (C) Synaptic silencing of αβ surface and γ neurons significantly reduces aversive olfactory 2-day LTM (*n* ≥ 8). (D) CAMEL-dependent expression of membrane-tagged GFP reveals sparse labeling of KCs. (E–F) Quantification of age-dependent CAMEL activity using cytosolic EGFP. (F) The number of innately labeled CAMEL neurons per brain hemisphere significantly increased from day 1 to day 7. No increase in CAMEL neuron number was observed between day 7 and day 14 (*n* ≥ 150, 6xCRE^AD^∩R21B06^DBD^ > EGFP). (G) In contrast to 1-day and 7-day-old flies, in 14-day-old flies the right hemisphere is more likely to contain more CAMEL cells (*n* ≥ 10, 6xCRE^AD^∩R21B06^DBD^ > EGFP). (H) Using constitutive labeling of CAMEL neurons, the number of labeled KCs significantly increased in 14-day or 21-day-old flies compared with 7-day-old flies (*n* ≥ 14, UAS-Flp/13xLexAopCD8GFP; 6xCRE^AD^,Brp-FRTstopFRT-V5-2A-LexA/R21B06^DBD^). (I) Flies raised in a food-odor–reduced environment displayed reduced CAMEL activity (*n* ≥ 172, 6xCRE^AD^∩R21B06^DBD^ > EGFP). Bars and horizontal lines represent mean ± SEM. For box blot: line, median; box, 75th–25th percentiles; whiskers, minimum to maximum. Scale bars correspond to 20 μm. Asterisks indicate significant differences between relevant groups (*P < 0.05, **P < 0.01, ***P < 0.001, ANOVA [C, H], Kruskal-Wallis [F], Mann-Whitney or unpaired *t* test [G], Mann-Whitney [I]). CAMEL, CRE-activity–dependent memory engram label; CRE, cAMP response element; CREB2, cAMP response element binding protein 2; EGFP, enhanced green fluorescent protein; Flp, Flippase; KC, Kenyon cell; LTM, long-term memory; SEM, standard error of the mean; UAS, upstream activating sequence.

To enable specific labeling and manipulation of olfactory LTM-encoding KCs, we developed a CREB reporter tool based on the split-Gal4 system. We utilized the conserved CRE sequence to control expression of the split-Gal4 activation domain (AD) (S1A Fig). We first tested whether reporter gene activation depended on the number of CRE elements (1, 3, 6, or 9) and observed the most efficient activation pattern for a 6xCRE construct (S1B Fig). To restrict activity to the MBs, we used the MB enhancer line R21B06^DBD^ that expresses the split-Gal4 DNA binding domain (DBD) in αβ surface and γ KCs (Fig 1B, elav^AD^∩R21B06^DBD^). We chose these 2 subpopulations of KCs for our analysis for two reasons: first, the synaptic output of αβ surface but not αβ core neurons is required for aversive LTM recall [9] and reduction of CREB activity in γ KCs was shown to decrease aversive LTM expression [51]. Second, although α’β’ neurons—the third major subtype of KCs—are essential for the recall of short-term memories (STMs) and anesthesia-resistant memories (ARMs), they are dispensable for LTM [57,58]. Consistent with these prior reports, expression of the neuronal silencing reagent tetanus toxin (TNT^G^) in R21B06 neurons (elav^AD^∩R21B06^DBD^) significantly decreased aversive 2-day LTM performance compared to controls (Fig 1C, see S4A Fig for odor and shock acuity controls). Thus, the combination of R21B06^DBD^ and 6xCRE^AD^ should report KC-specific activation of the cAMP/CREB-signaling pathway and thereby provide a CRE-activity–dependent memory engram label (CAMEL) (Fig 1A). Analysis of endogenous CAMEL activity in KCs using a membrane-bound green fluorescent protein (GFP) reporter (6xCRE^AD^∩R21B06^DBD^ > mCD8-GFP) revealed labeling of a small number of individual KCs (Fig 1D). Quantification of cell body labeling in large populations of flies using a cytosolic enhanced GFP (EGFP) reporter (6xCRE^AD^∩R21B06^DBD^ > EGFP) demonstrated sparse, nonstereotypic and variable activity of the CAMEL tool (Fig 1E and 1F). We observed a significant increase in CAMEL activity in 7-day-old flies compared to 1-day-old flies (Fig 1F). This indicates that, during early adulthood, natural sensory experiences may contribute to an increase in CAMEL-tool activity. In contrast, in 14-day-old flies we did not observe any further increase in the number of labeled KCs (Fig 1F). However, these flies displayed a significant labeling bias between left and right brain hemispheres that was not present in either 1-day or 7-day-old flies (Fig 1G). This change of brain hemisphere labeling preference between 7-day and 14-day-old flies, despite constant cell numbers, points to a dynamic labeling feature of our CAMEL tool that would be consistent with the reported transient nature of CREB2 activity during memory formation and maintenance (approximately 4 days) [55,59]. We addressed this experimentally via CAMEL-mediated expression of a Flippase (Flp) that constitutively activates a flip-out–dependent LexA/LexAop reporter system and thus results in permanent labeling of CAMEL neurons. In these CAMEL flip-out flies, we observed a significant increase in labeled KCs in 14-day and 21-day-old flies compared to 7-day-old flies (Fig 1H, S1C Fig). These results demonstrate that the CAMEL tool remains active after day 7 and has the potential to label the entire pool of KCs, while CAMEL activity within individual KCs is transient.

Next, we aimed to explore the potential sensory experience-dependent characteristics of our CAMEL tool. Therefore, we raised flies in an odor-free environment, an approach that has been previously used to decrease activity in the olfactory pathway [60]. Reduction of food-associated odors resulted in a significant decrease in labeling efficacy of KCs indicating that food-odor associations may contribute to endogenous CAMEL activity (Fig 1I). These data show that innate CAMEL activity increases in the first week of adult life in a sensory experience-dependent manner and thus potentially reflects natural memory formation.

### CAMEL reflects cAMP/CREB-signaling activity

**Fig 2:**
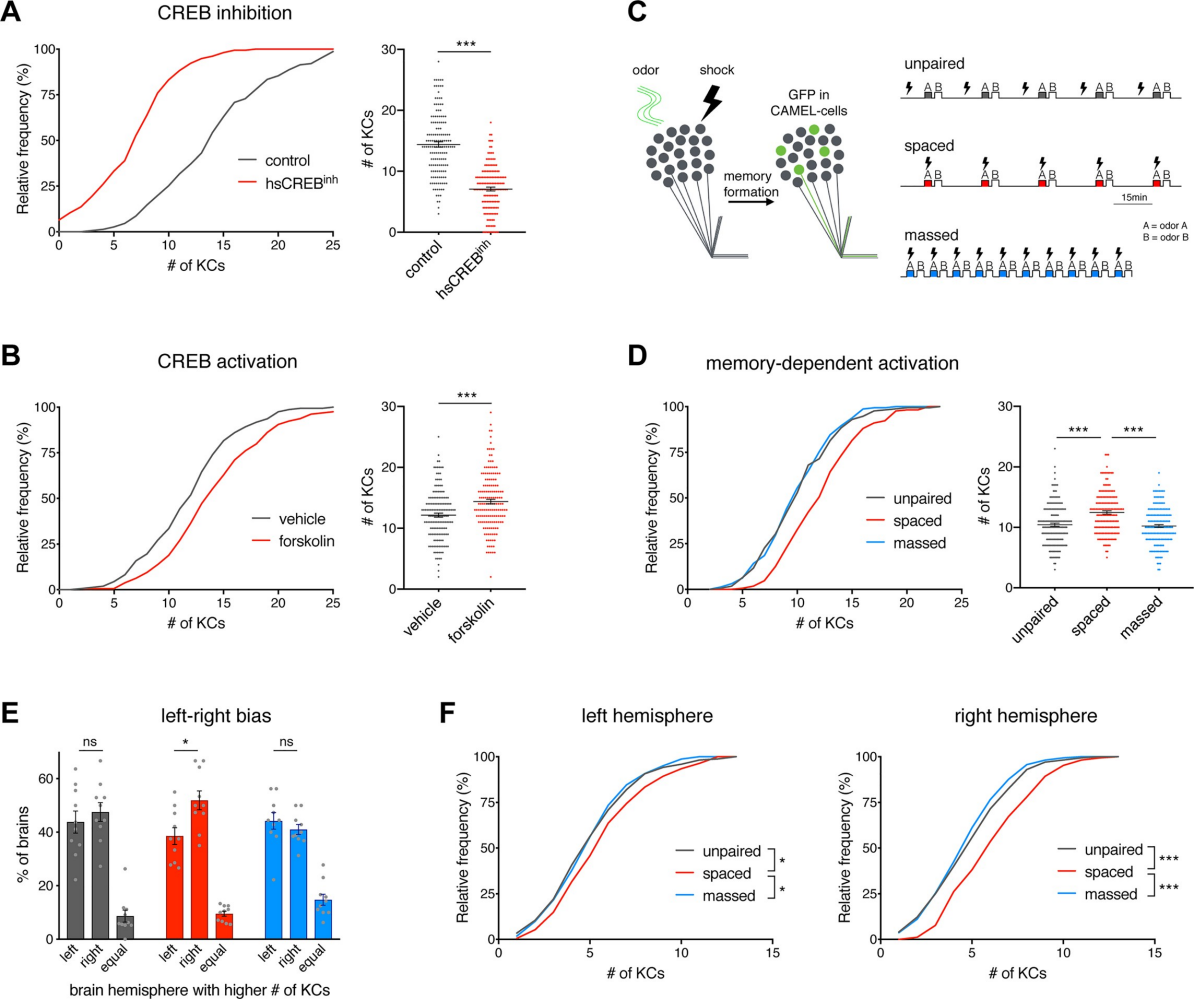
CREB2 and LTM-dependent CAMEL activity. (A) Expression of an inhibitory CREB2 isoform (CREB2b) significantly decreased the number of labeled CAMEL neurons (*n* ≥ 143). (B) In contrast, elevated cAMP signaling via forskolin feeding increased labeling of CAMEL neurons (*n* ≥ 158). (C) Schematic drawing of experimental principle and conditioning protocols. (D) Only spaced paired conditioning resulted in increased CAMEL activity (*n* ≥ 162). (E) In contrast to control groups (unpaired trained, grey bars; massed trained, blue bars), spaced trained flies (red bars) show a bias towards increased labeling in the right hemisphere (*n* ≥ 10). (F) Analysis of both brain hemispheres separately revealed that LTM formation protocols led to increased CAMEL activity in both the left and the right brain hemispheres, with stronger effects in the right brain hemisphere (*n* ≥ 162). (A–F; 6xCRE^AD^∩R21B06^DBD^ > EGFP). Bars and horizontal lines represent mean ± SEM. Asterisks indicate significant differences between relevant groups (*P < 0.05, ***P < 0.001, Mann-Whitney [A, B], Kruskal-Wallis [D, F], or unpaired *t* test [E]). CAMEL, CRE-activity–dependent memory engram label; CRE, cAMP response element; CREB2, cAMP response element binding protein 2; EGFP, enhanced green fluorescent protein; KC, Kenyon cell; LTM, long-term memory; SEM, standard error of the mean.

To demonstrate that the observed alterations in labeling efficacy are dependent on changes in cAMP/CREB-signaling pathway activity, we expressed an inhibitory isoform of CREB2 [5] independent of the Gal4-upstream activating sequence (UAS) system using a heat shock promoter. Expression of the CREB2 inhibitor resulted in a significant decrease in CAMEL activity compared to control flies exposed to the same heat shock protocol (Fig 2A). In addition, pharmacological up-regulation of the cAMP/CREB pathway by feeding flies the adenylyl cyclase activator forskolin [61] led to a significant increase in the number of CAMEL-positive KCs (Fig 2B). Together, these results demonstrate that the CAMEL tool reflects alterations in cAMP/CREB signaling.

### LTM-dependent labeling of potential engram cells

Based on these findings, we hypothesized that our CAMEL tool may enable selective tagging of KCs that undergo CREB2-mediated plasticity during aversive olfactory LTM formation. To address this, we exposed sibling flies to 3 different training protocols, which differ in timing but share an identical sensory experience: unpaired, spaced, and massed training (Fig 2C). In contrast to unpaired and massed training, only spaced training induces a CREB2 and protein-synthesis–dependent form of LTM [5,62]. To assess CAMEL activity in response to LTM formation, we trained sibling flies using a spaced, massed, or unpaired protocol and analyzed the number of CAMEL-positive KCs 2 days later. The immunohistochemical analysis revealed a significant increase in CAMEL-positive KCs in the spaced trained group compared to the unpaired or massed trained groups (Fig 2D). This sensitivity of the CAMEL tool to specific training experiences indicates that it labels KCs with high CREB2 activity during protein-synthesis–dependent LTM formation. Interestingly, in the spaced trained group, we observed a right-to-left brain hemisphere labeling bias (Fig 2E) that resembled the effect observed in 14-day-old flies (Fig 1G). To address whether LTM formation preferentially induces CAMEL activity in the right brain hemisphere, we analyzed the hemisphere-specific CAMEL-cell distribution. We observed significant increases in CAMEL cells in response to LTM formation in both the left and the right hemisphere but more pronounced effects in the right hemisphere (Fig 2F, S2A Fig and S2B Fig).

### Natural recall cue elicits neuronal activity in CAMEL neurons

**Fig 3:**
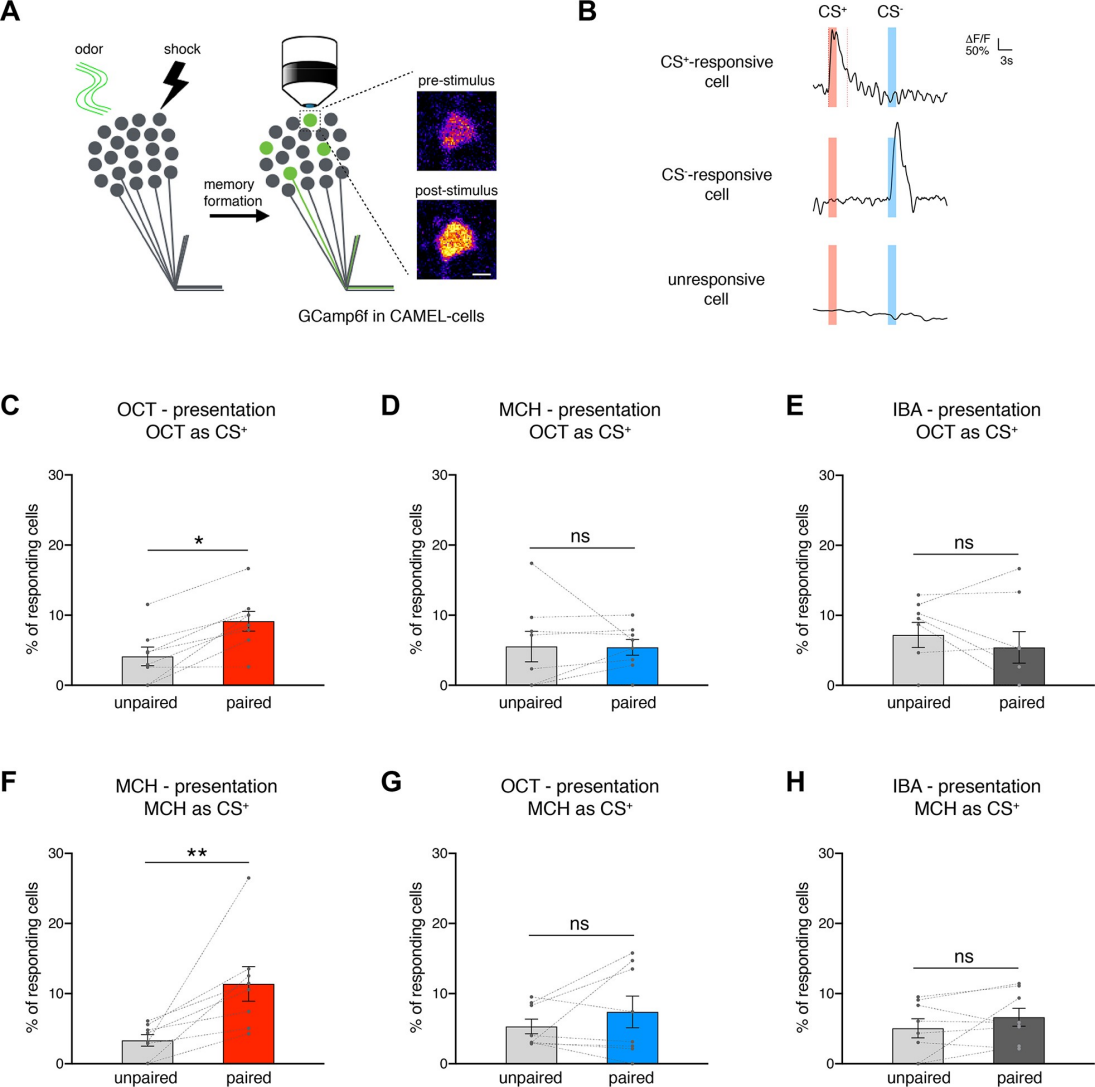
Natural recall cue activates CAMEL cells in vivo. (A) Schematic of the experimental design. Exemplary KC soma in vivo recording shows increased GCaMP6f response after presentation of an olfactory cue (OCT) to the fly’s antennae. (B) Three GCaMP6f example traces of individual KCs (upper trace from example in panel A). Red (CS^+^, OCT) and blue (CS^−^, MCH) bars indicate odor presentation time window, and red dashed lines indicate the time window for analysis (0–4.5 seconds post stimulus). (C–E) LTM conditioning (OCT as CS^+^) significantly increased the percentage of CAMEL cells responding to OCT (CS^+^, panel C) compared to control flies (unpaired training) 3–4 days after conditioning (*n* ≥ 8; 10–55 cells per *n*; total ≥ 249 cells). No difference was observed for MCH-responding cells (CS^−^, panel D) or cells that respond to a training-unrelated odor (IBA, panel E). (F–H) Conditioning using reversal of odors (MCH as CS^+^ and OCT as CS^−^) again resulted in an increased percentage of CAMEL cells responding to the CS^+^ (MCH, panel F) but not to the CS^−^ (OCT, panel G) or IBA (panel H) (*n* ≥ 8; 19–47 cells per *n*; total ≥ 240 cells). (C–H) All data were acquired 3–4 days after training, and each *n* represents data from an independent round of conditioning (6xCRE^AD^∩R21B06^DBD^ > GCaMP6f, tdTomato). Bars represent mean ± SEM (lines connect data points collected in parallel on the same day). Scale bar corresponds to 2.5 μm. Asterisks indicate significant differences between relevant groups (*P < 0.05, **P < 0.01, unpaired *t* test). CAMEL, CRE-activity–dependent memory engram label; CRE, cAMP response element; CS, conditioned stimulus; GCaMP,; IBA, Isobutyl-acetate; KC, Kenyon cell; LTM, long-term memory; MCH, 4-Methyl-cylohexanol; ns, not significant; OCT, 3-Octanol; SEM, standard error of the mean.

We next tested whether CAMEL neurons specifically respond to the behaviorally relevant conditioned stimulus (CS^+^) used in the conditioning assay. To directly monitor neuronal activity, we expressed the calcium reporter GCaMP6f in CAMEL neurons and tested odor responses in vivo 3 to 4 days after either paired or unpaired LTM conditioning (Fig 3A and Fig 3B). In flies that were mock trained with unpaired stimuli, odor presentations elicited Ca^2+^ responses in approximately 5% of CAMEL-GCaMP6f-expressing neurons (4.1% 3-Octanol [OCT], 5.5% 4-methylcylohexanol [MCH]; Fig 3C and Fig 3D). These values are consistent with imaging of all R21B06-positive KCs (elav^AD^∩R21B06^DBD^; 5.8% OCT, 3.9% MCH; S3E Fig, S3F Fig and S3G Fig) and prior functional analyses of the entire KC population demonstrating selective odor activity in approximately 5% of KCs [26,28]. This indicates that innately labeled CAMEL neurons are randomly tuned to olfactory cues used in this study. In contrast, paired conditioning using OCT as CS^+^ and MCH as CS^−^ resulted in a significant increase in the percentage of CAMEL neurons responding to OCT (Fig 3C) but not to MCH (Fig 3D) or to a third, training-independent odor (Isobutyl-acetate [IBA]; Fig 3E). Similarly, when flies were trained using MCH as CS^+^ and OCT as CS^−^, the percentage of cells that responded to MCH was significantly increased (Fig 3F), while the response to OCT (Fig 3G) or IBA (Fig 3H) remained unchanged. When we quantified the number of animals displaying CS^+^-responsive CAMEL-GCaMP6f-expressing neurons, we also observed a significant increase in the paired conditioned cohorts (S3A Fig and S3C Fig). This demonstrated that the increase of CS^+^-responsive cells was the result of changes in many animals. For the CS^−^, we did not observe significant differences between the paired and unpaired trained groups (S3B Fig and S3D Fig). Thus, LTM conditioning significantly increased the percentage of CAMEL neurons responding to the natural recall cue.

### Synaptic output of CAMEL neurons is required for LTM

**Fig 4:**
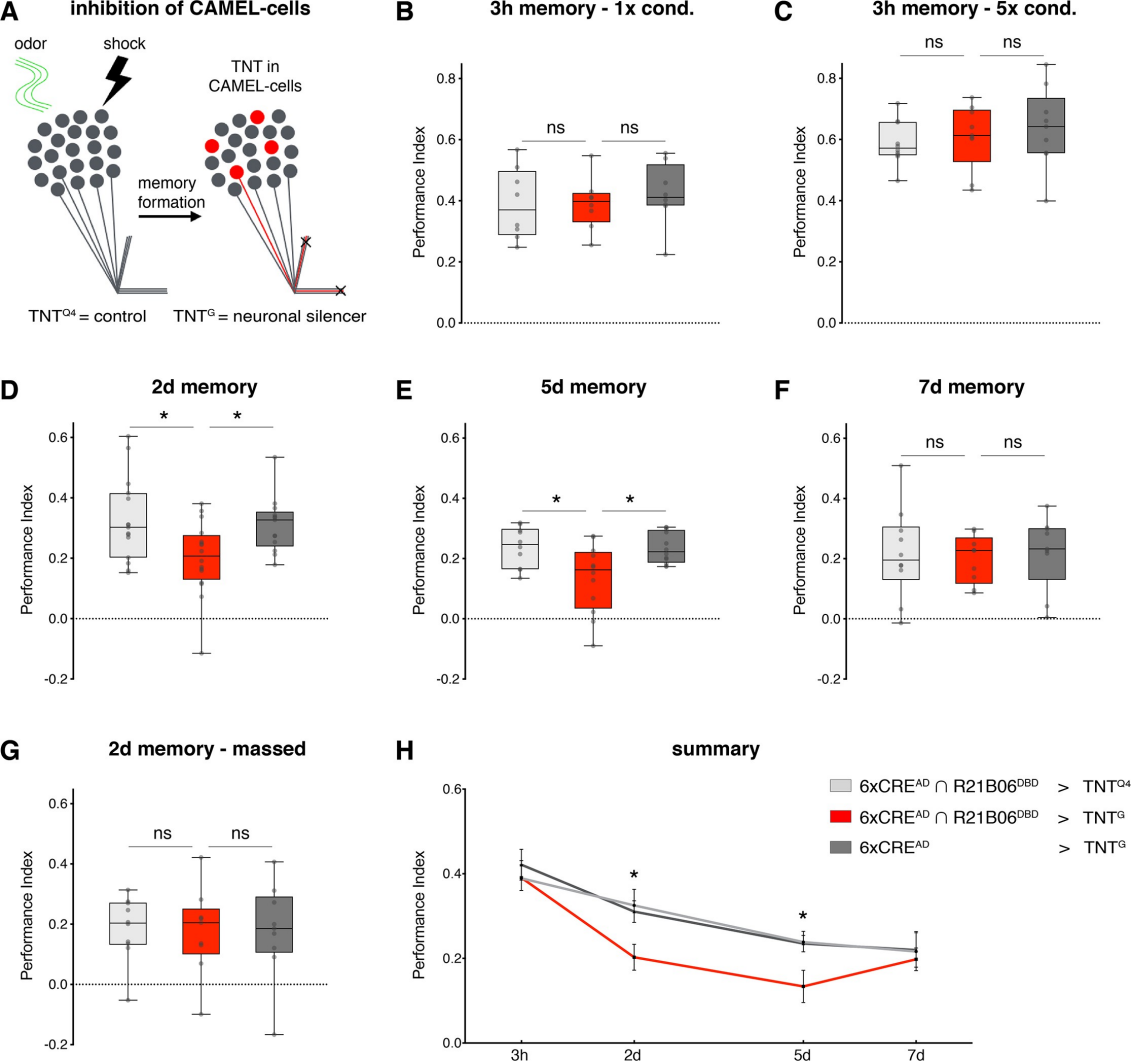
CAMEL neurons are required for LTM. (A) Schematic of the experimental design. CAMEL neurons are silenced by TNT expression to determine their requirement for memory expression. (B–C) Three-hour memory performances of control and experimental genotypes were indistinguishable after 1 (panel B) or 5 (panel C) rounds of spaced training. (D–E) Flies expressing active TNT in CAMEL neurons showed a significant reduction in aversive olfactory memory performance 2 days (panel D) and 5 days (panel E) after spaced training. (F) Seven days after spaced conditioning, the experimental genotype had no defect in memory performance. (G) The experimental genotype displayed normal ARM memory performance 2 days after massed training. (H) Summary of the data. For box blots: line, median; box, 75th–25th percentiles; whiskers, minimum to maximum (*n* ≥ 8). Data in panel H represent mean ± SEM. Asterisks indicate significant differences between relevant groups (*P < 0.05, ANOVA). ARM, anesthesia-resistant memory; CAMEL, CRE-activity–dependent memory engram label; CRE, cAMP response element; LTM, long-term memory; ns, not significant; SEM, standard error of the mean; TNT; tetanus toxin.

We next assessed the functional relevance of the CAMEL-tagged KC population for aversive olfactory LTM. We used the CAMEL tool to express the neuronal silencing tool TNT^G^ in potential KC engram neurons to inhibit their synaptic output (Fig 4A). This manipulation did not impair odor or shock acuity (S4A Fig). Three hours after training, when memory is still protein synthesis independent (mid-term memory [MTM]) [49,63], memory recall of the experimental genotype was not significantly different from parental controls or from controls expressing an inactive form of TNT (TNT^Q4^) regardless of the applied training protocol (single round of conditioning, Fig 4B; or 5× spaced conditioning, Fig 4C). Similarly, ARM performance 2 days after massed training was indistinguishable between the 3 genotypes (Fig 4G). These data demonstrate that inhibition of innate CAMEL neurons does not affect sensory perception or MTM/ARM formation and recall. Importantly, 2 days after spaced conditioning, a significant reduction in LTM performance was observed in TNT^G^-expressing animals compared to controls (Fig 4D). This significant decrease in memory expression was still evident 5 days after spaced training (Fig 4E). Strikingly, 7 days after training, memory performance was indistinguishable from control levels (Fig 4F and Fig 4H). After correcting for natural forgetting (see Materials and methods), the memory performance of the experimental genotype on day 7 was significantly higher compared to day 5 (S4B Fig, S4C Fig and S4D Fig). Thus, CAMEL-mediated neuronal silencing only transiently blocked memory. These data are consistent with the transient GFP-labeling characteristics of our CAMEL tool (Fig 1) and with the observation that memory maintenance becomes CREB2 independent at late stages of aversive olfactory LTM [55]. Based on these results, we conclude that silencing of LTM-relevant CAMEL neurons did not disrupt LTM formation or maintenance but selectively impaired memory recall at days 2 and 5 due to a selective and transient block of synaptic output of CAMEL neurons. Together, our data show that the CAMEL tool enables manipulation of neurons functionally required for LTM expression.

### Artificial activation of CAMEL neurons evokes aversive memory recall

**Fig 5:**
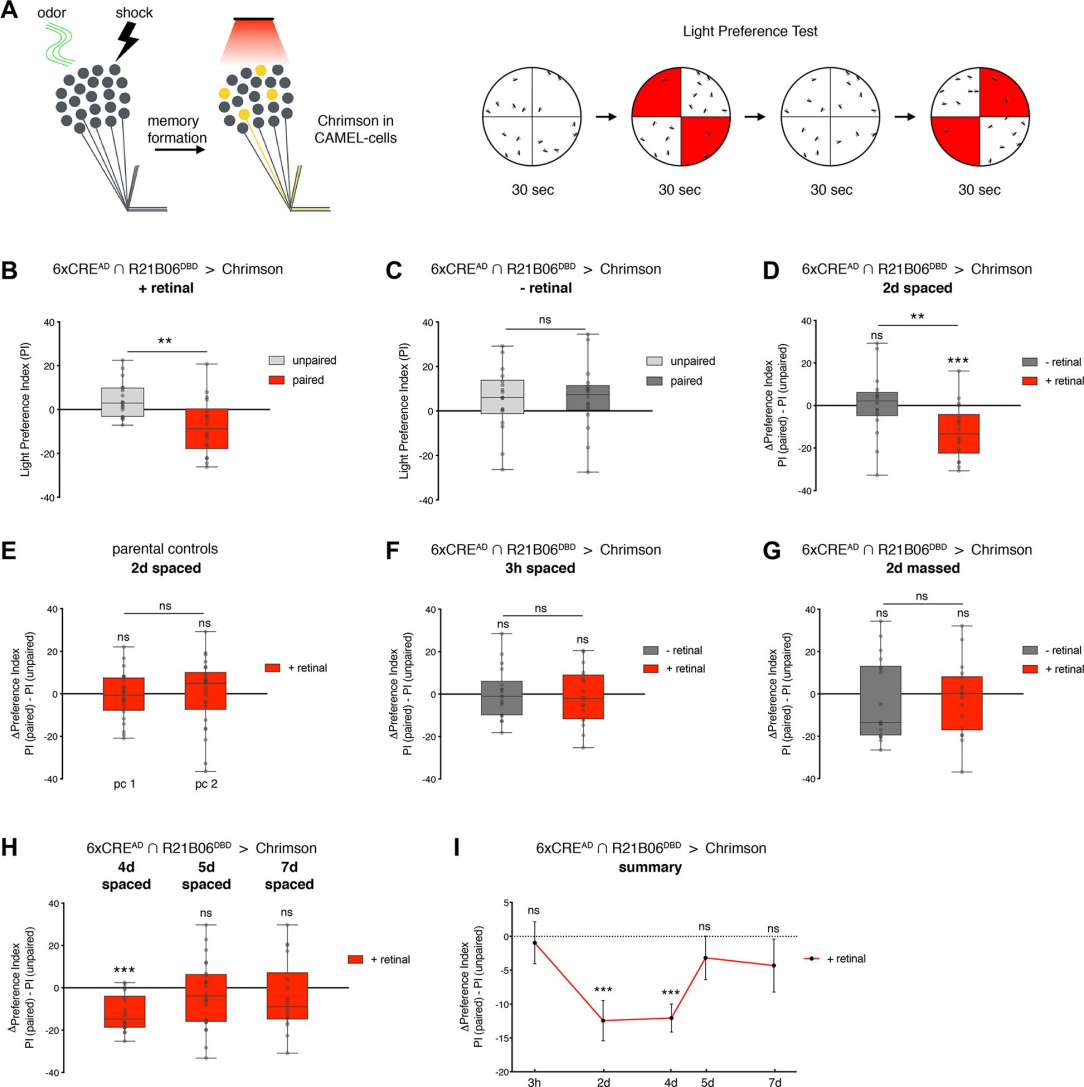
Artificial reactivation of CAMEL neurons elicits aversive behavior. (A) Schematic drawing of the experimental setup and the light preference test. (B) Two days after spaced training, paired conditioned flies significantly avoid red light quadrants compared to unpaired controls. (C) This aversive behavior depends on feeding of the essential Chrimson co-factor retinal. (D) The difference between PIs of paired and unpaired groups (ΔPI, from panels B–C) represents total light avoidance (−12.44 ± 2.99 for retinal-fed flies 2 days after training) and is significantly different from 0 and significantly different between retinal- and non–retinal-fed cohorts. (E) ΔPIs of parental control genotypes that were subjected to identical training and testing protocols including retinal feeding were not significantly different from 0 (pc 1: UASChrimson/+; 6xCRE^AD^/+ and pc 2: R21B06^DBD^/+). (F–G) No change in light avoidance between paired and unpaired trained groups was observed 3 hours after spaced training (panel F) or 2 days after massed training in the presence or absence of retinal (panel G). (H) Four days after spaced training, the experimental group showed a ΔPI that significantly differs from 0 (−12.06 ± 2.06 for retinal-fed flies 4 days after training). No significant change in light preference was observed 5 days or 7 days after training. (I) Summary of the data. For box plots: line, median; box, 75th–25th percentiles; whiskers, minimum to maximum (*n* ≥ 18). Data in panel I represent mean ± SEM. Asterisks indicate significant differences between relevant groups or 0 (**P < 0.01, ***P < 0.001, unpaired and one-sample *t* test). CAMEL, CRE-activity–dependent memory engram label; CRE, cAMP response element; ns, not significant; pc, parental control; PI, Preference Index; SEM, standard error of the mean; UAS, upstream activation sequence.

Finally, we tested whether CAMEL neurons are not only required but also sufficient for memory recall. In an optogenetic preference assay in which flies can freely choose between dark- or red-light–illuminated areas, we artificially reactivated CAMEL neurons using the red-light–sensitive Channelrhodopsin variant Chrimson [64] after aversive olfactory conditioning (Fig 5A). Consistent with prior studies [36], this assay is able to resolve whether flies prefer or avoid activation of Chrimson-expressing neurons by assessing their position relative to the red-light–illuminated areas (S5A Fig). After unpaired training, flies expressing Chrimson in CAMEL neurons showed a slight, but not significant, preference towards the red light (Fig 5B). In contrast, 2 days after paired aversive conditioning, flies displayed an aversion to the red light resulting in a negative light Preference Index (PI) that was significantly different from the unpaired control group (Fig 5B). This effect required feeding of the Chrimson co-factor all-trans-retinal between training and testing (Fig 5C and Fig 5D) demonstrating that Chrimson-mediated activation of conditioning-dependent CAMEL-positive KCs elicited aversive behavior (ΔPI −12.44 ± 2.99) (Fig 5D). In comparison, we did not observe any significant differences in parental controls that underwent the same protocol, confirming that retinal feeding had no influencing effects (Fig 5E). Furthermore, we did not observe any significant effects when testing flies 3 hours after spaced or 2 days after massed training (Fig 5F and Fig 5G). To test the perdurance of the CAMEL-mediated light-evoked aversive behavior, we repeated this experiment 4, 5, and 7 days after training. Interestingly, we observed a significant increase in red light aversion 4 days but not 5 or 7 days after conditioning (Fig 5H and Fig 5I). These data demonstrate that reactivation of conditioning-dependent tagged CAMEL neurons is sufficient to reinstate aversive behavior phenocopying LTM recall. The differences in CAMEL off-kinetics between our inhibition (Fig 4H) and activation experiments (Fig 5I) are likely due to differences of the Chrimson and TNT tools in expression levels, potency, or turnover rates. Together, our observational, loss-, and gain-of-function data are consistent with CAMEL-tagged neurons representing *Drosophila* engram cells.

## Discussion

In this study, we established a novel tool enabling genetic access to KCs encoding individual aversive olfactory LTMs in *Drosophila*. In mice and flies, prior studies utilized expression of the immediate-early gene c-Fos to successfully tag populations of neurons containing memory engram cells [42–45]. Here, we aimed to generate a tool enabling specific tagging of LTMs. Therefore, we designed the CAMEL tool to directly report CREB2 activity that is essential not only for induction but also for the consolidation and maintenance of LTMs [5,52,55,56]. Thus, in contrast to the activity-based methods, this tool should be suitable to endogenously tag, monitor, and manipulate engram cells over prolonged periods of engram persistence. In accordance with these features, we observed long-lasting LTM-specific CAMEL induction in CS^+^-activated cells. Silencing and activation experiments showed that these CAMEL-tagged cells are necessary and sufficient for aversive LTM expression. Importantly, in these experiments, CAMEL activity reflected the time frames of physiological CREB2-dependent LTM formation and maintenance [55,59]. This time course, together with the observation that innate CAMEL activity decreased upon food-associated odor deprivation, indicates that innate CAMEL activity may reflect natural memory formation. Variable CAMEL activity between flies may thus underlie the plasticity-driven interanimal differences in odor tuning previously observed at the level of MBONs [25]. In aged flies and upon LTM induction, we observed a significant right-to-left brain hemisphere bias of CAMEL tagging. While the relevance of this right-side bias for LTM remains to be explored, it is consistent with the observation that asymmetry of a central brain structure named asymmetric body is required for effective LTM performance [65]. It will be interesting to test how asymmetries of memory coding at the level of KCs may contribute to directional decision-making during olfactory 2-choice assays.

Our GFP- and GCaMP-based observational experiments revealed conditioning-dependent CAMEL activity in a relatively low number of cells in comparison to prior studies estimating putative engram cell number in *Drosophila* [45,66]. These studies utilized either artificial memory induction in randomly labeled KCs [66] or c-Fos-activity–based labeling [45] to estimate engram cell size shortly after training. Thus, differences in the technical approaches and in the time point of memory engram size evaluation likely contribute to these discrepancies. Furthermore, due to constraints of the CAMEL design, we likely underestimate the actual number of KCs encoding individual LTMs. Because the CAMEL tool reports LTM-dependent CREB2 activity without amplification, our GFP- and GCamP6f-based observational assays likely fail to capture all engram cells. The enzymatic and channel properties of TNT and Chrimson in our functional assays potentially modulated the activity of engram cells not identified in the observational assays. Second, in mice, a preferential recruitment of CREB-overexpressing cells into the memory engram has been described and used to tag engram cells (memory allocation) [67,68]. Accordingly, in our system, endogenously tagged CAMEL cells might have been allocated to the engram and thereby potentially contributed to memory performance in the activation and silencing assays. Finally, it is important to note that our evaluation of aversive LTM is based on population analyses that currently prevent conclusions regarding successful LTM formation in individual flies.

At a circuit level, the impairment of 2- and 5-day memory after CAMEL-cell silencing demonstrated that only a small subset of KCs is required for *Drosophila* LTM expression. The intact memory at day 7 despite multiple days of engram cell silencing argues that KC engram output is dispensable for LTM storage. Recent studies demonstrated that aversive STMs are encoded by depression of KC > MBON^approach^ synapses [33,34,36]. As a consequence, CS^+^ exposure during memory recall leads to a differential activation of approach and avoidance output channels resulting in net avoidance (“balance” model [38,39]). Our data are in general agreement with such a balance model for LTM; silencing of CAMEL neurons selectively eliminates KCs with biased output from the circuitry and thus disrupts memory recall. Optogenetic activation of these biased KCs induces aversive behavior via a preferential activation of aversive MBONs indicating that compartment-specific LTM-dependent synaptic changes [34,35] uphold artificial reactivation. Because silencing of individual aversive MBONs does not impair memory performance [36], it is likely that engram KCs simultaneously activate multiple aversive MBONs to induce aversive behavior. Support for such a model is provided by the EM reconstruction that demonstrated extensive synaptic connectivity between individual KCs and all MBONs in analyzed MB compartments [40]. We find that the effects on memory are mediated by a surprisingly small number of KCs in accordance with the observation that insect KC > MBON synapses are strong [69]. Moreover, the high number of KC > KC synapses [40,41] and converging KC > MBON synapses (or rosettes [40]) may provide amplification mechanisms to enhance the impact of individual KCs on circuit output and behavior.

Together, our study advances our understanding of memory circuitry and function and provides a novel entry point to unravel the cellular and synaptic changes underlying LTM. Because CREB represents a general, evolutionary conserved biochemical LTM switch, CAMEL might be applicable to a wide range of memory studies in different systems.

## Materials and methods

### Fly stocks

Flies were kept on standard fly food at 22°C and 70% humidity unless otherwise noted. Flies subjected to aversive conditioning and GFP-labeling experiments were reared and collected at 18°C and 70% humidity in standard fly bottles. One day before experiments, flies were split into standard fly vials and shifted to 22°C.

Fly stocks used in this study are as follows: *elav*-split-Gal4^DBD^ [70] (BDSC23867), *elav*-splitGal4^AD^ [70] (BDSC23868), 2xUAS-*EGFP* [71] (BDSC 23867), *R21B06*-splitGal4^DBD^ [18] (MB364B), 10xUAS-*mCD8GFP* [72] (BDSC 32186), UAS-*TNT*^*G*^ [73] (BDSC 28838), UAS-*TNT*^*Q4*^ [73] (BDSC 28839), UAS-*Flp* [74] (BDSC 4539), 13xLexAop-*mCD8GFP* [72] (BDSC 32205), *Brp-FRTstopFRT-V5-2A-LexA* [75], hs-*CREB2b* [5] (17–2), 20xUAS-*Chrimson*^*VENUS*^ [64] (BDSC 55135), Gr66a-Gal4 [76], MB077B [18], 20xUAS-*GCaMP6f* [77] (BDSC 42747), and 10xUAS-*tdTomato*^*myr*^ [72] (BDSC 32223). BDSC stocks were obtained from the Bloomington Drosophila Stock Center, Indiana University, Bloomington.

### Molecular biology

For the generation of the CAMEL tool, we annealed complementary DNA sequences (Microsynth AG, Balgach, Switzerland) containing restriction sites and varying numbers of CRE sites (S3 Table). The double-stranded DNAs were inserted into pENTR vectors (Thermo Fisher, Waltham, MA) using restriction ligation. CRE constructs were then cloned into the pBPp65ADZpUw vector [72] via gateway cloning (Thermo Fisher, Waltham, MA). All cloning steps were verified by sequencing. Transgenic flies were generated using the attP2 landing site using the phi-C31 system [78].

### Aversive olfactory conditioning

Aversive conditioning was performed as described by Tully and colleagues [49] under weak red light at 20°C to 22°C and 75% humidity using 2- to 7-day-old flies. We used a custom-made conditioning device allowing parallel training of 8 groups of flies (details available on request). Flies were loaded into custom-made copper grid tubes and exposed to a constant air stream (750 mL/min). To expose flies to odors, the air stream was odorized via air suction (750 mL/min) through odor containing mineral oil (1:250, Thermo Fisher, Waltham, MA, CAS #8042-47-5). After 2-minute resting periods (air only), flies were simultaneously exposed to CS^+^ and electric shocks (twelve 1.5-second 90 V shocks with 3.5-second intervals) for 60 seconds. Subsequently, flies were exposed to air for 45 seconds and then to the CS^−^ for 60 seconds. This training cycle was repeated 5 times in 15-minute intervals for spaced training and 10 times in 45-second intervals for massed training. For unpaired conditioning, flies received 60-second electric shocks (twelve 1.5-second 90 V shocks with 3.5-second intervals) in the absence of odor presentation. After 5-minute recovery in constant airflow, flies were exposed to 60 seconds of the first odor, 45 seconds of air, and 60 seconds of the second odor. Five training cycles were performed in 9-minute intervals to keep the overall duration of the experiment identical to the spaced training protocol. Flies were kept at 18°C for the indicated amount of time before memory test in the T-maze [49]. For testing, flies were brought to the decision point of a T-maze at which they could choose for 2 minutes between CS^+^ and CS^−^ (1:1,000) containing arms. Odors used for conditioning were 4-MCH (Merck, Darmstadt, Germany, CAS #589-91-3) and OCT (Merck, Darmstadt, Germany, CAS #589-98-0). For each experiment, 2 groups of flies were conditioned with reversed odor pairings such that each odor served one time as CS^+^ and one time as CS^−^. For each group, Performance Indices (PerIs) were calculated as follows:

$$PerI=\frac{\#of\;flies\;(CS-)-\#of\;flies\;(CS+)}{\#of\;flies\;(CS+)+\#of\;flies\;(CS-)}*100$$

Averaging the 2 PerIs of reciprocal groups results in the final PerI. For Chrimson experiments, flies were conditioned in parallel using either the paired (spaced) or unpaired protocol and kept at 18°C for the indicated amount of time until testing. The unpaired group in Fig 5G was shocked only once 5 minutes before odor presentations. Flies were kept in the dark on all-trans-retinal containing food (0.5 mM, Sigma-Aldrich) for 2 days before testing (see “Light preference test”). For GCaMP6f experiments, flies were paired (spaced) and unpaired conditioned in parallel as described above and subsequently stored at 18°C for 3 to 4 days before performing live imaging (see “In vivo calcium imaging”).

For direct comparison of memory performance across days (S4B Fig, S4C Fig and S4D Fig), we calculated a forgetting factor (F) as follows:

$$F=\frac{\Delta PerI(5d-7d\;memory\;of\;control1)+\Delta PerI(5d-7d\;memory\;of\;control2)}{2}$$

### Odor acuity and shock responsiveness

Odor acuity and shock responsiveness was performed in a T-maze as described by Keene and colleagues [79]. For odor acuity, flies were given 2 minutes to choose between an odor (OCT or MCH) diluted in mineral oil (1:1,000) and pure mineral oil. To test shock responsiveness, flies could freely choose for 60 seconds between a voltage-carrying shock tube (twelve 1.5-second 90 V shocks with 3.5-second intervals) and a nonconnected shock tube under constant airflow. Avoidance scores (ASs) were calculated as follows:

$$AS=\frac{\#of\;flies\;(experimental\;tube)-\#of\;flies\;(control\;tube)}{\#of\;flies\;(experimental\;tube)+\#of\;flies\;(control\;tube)}*100$$

Experimental tubes (odor, shock) and control tubes (no odor, no shock) were alternately placed into the right and left slot of the T-maze.

### GFP-labeling experiments

For GFP-labeling experiments after conditioning, 2- to 5-day-old flies were conditioned as described and stored at 22°C for 2 days before brain dissection. For odor-reduced environment experiment, late pupae were washed and transferred to vials containing odor-reduced food (1M D-Sorbitol, 2% Agarose) or standard food at 22°C. Three- to 4-day-old flies were dissected and analyzed for GFP expression. In forskolin feeding experiments, 1- to 3-day-old flies were transferred onto food containing 10 μM forskolin (Enzo Life Sciences GmbH, Lörrach, Germany) or onto standard food containing identical volumes of vehicle (PBS) and stored for 3 to 4 days at 22°C before dissection. For heat shock-CREB2b experiments, 1- to 3-day-old flies were subjected to 3 heat shocks (37°C water bath, 30 minutes) in 24-hour intervals prior to dissection.

To quantify GFP-labeling experiments, cell bodies of MB neurons were counted using a PL APO 20× oil immersion objective (Leica Microsystems GmbH, Wetzlar, Germany).

### Immunohistochemistry and microscopy

Dissection and antibody labeling was performed as described previously [80]. Briefly, flies were incubated in 4% PFA + 0.2% Triton-X 100 (Merck, Darmstadt, Germany) for 3.5 hours at 4°C and then washed for 3 × 30 minutes in PBS + 0.2% Triton-X 100 at room temperature prior to brain dissection. Dissected brains were incubated in primary antibodies for 2 to 5 days and in secondary antibodies for 2 days at 4°C. Afterwards, brains were washed for at least 3 × 30 minutes. The following antibodies were used in this study: rabbit anti-GFP (A6455, Thermo Fisher, Waltham, MA) (1:1,000); rabbit anti-Dlg [81] (1:30,000); rat anti-CD8a (MCD0800, Thermo Fisher, Waltham, MA) (1:500); mouse anti-Brp (nc82, Developmental Studies Hybridoma Bank, Iowa City, Iowa) (1:200); rabbit anti-dsRed (632496, Takara, Kyoto, Japan) (1:500); mouse anti-V5 (960–25, Thermo Fisher, Waltham, MA) (1:500); and Alexa488, 568, and 647 coupled secondary antibodies (Thermo Fisher, Waltham, MA) (1:1,000). Brains were mounted in Vectashield (Vector Laboratories, Burlingame, CA). Images were captured using a Zeiss LSM 700 laser scanning confocal microscope with a 25× (NA 0.8) or 40× (NA 1.3) oil immersion objective (Carl Zeiss Microscopy GmbH, Jena, Germany). Images were processed using Imaris (Bitplane AG, www.imaris.oxinst.com) and Adobe Photoshop software (Adobe; www.adobe.com).

### Light preference test

A light preference test was performed in a custom-built choice arena as described by Aso and colleagues and by Klapoetke and colleagues [36,64]. Briefly, approximately 20 flies were placed into the arena under constant airflow (150 mL/min) at 20°C and 60% humidity. The arena was controlled using Arduino UNO and Matlab to expose the flies to the following light exposure protocol: 30 seconds of no light, 30 seconds of red light in 2 quadrants, 30 seconds of no light, and 30 seconds of red light in the other 2 quadrants. The amount of 20 μW/mm^2^ red light (627 nm) intensity was used for all experiments. Flies were monitored and counted using infrared light and an infrared camera (Rasperry Pi Camera V2.1). Light PI of 1 time point was calculated as follows:

$$PI=\frac{\#of\;flies\;(illuminated\;quadrants)-\#of\;flies\;(dark\;quadrants)}{\#of\;flies\;(illuminated\;quadrants)+\#of\;flies\;(dark\;quadrants)}*100$$

For one experiment, the total PI is the average of 10 PIs from the last 5 seconds of the 2 light-on episodes. ΔPI represents the difference between the PI of the paired group minus the PI of the unpaired group.

### In vivo calcium imaging

Flies were anaesthetized on ice for 10 to 15 seconds and mounted on a custom-built chamber using paraffin wax (Merck, Darmstadt, Germany) and a dental waxing device. The head, proboscis, and legs were immobilized with wax, and the chamber was filled with ice-cold sugar-free HL3-like saline [82]. To access the brain, a small coronal window through the fly’s head cuticle was opened. Images were acquired using an Ultima Investigator Multiphoton Imaging System (Bruker, Billerica, MA) powered by Prairie View Imaging Software (Bruker, Billerica, MA) and a Chameleon Vision II excitation laser (Coherent, Palo Alto, CA) at 940 nm. Imaging was performed using a 20× water immersion objective (NA 1.0 XLUMPLFLN, Olympus, Tokyo, Japan). All images were uniformly acquired with a galvo scanner at a 16× optical zoom and a dwell time of 1.4–1.6 μs and were formatted to 256 × 256 pixels of 16 bit resulting in a 7 Hz frame rate.

For odor presentation, we used a custom-built olfactory presentation device similar to previously described setups [83]. We used manual airflow meters (Cole Parmer, Vernon Hills, IL) to adjust a carrier stream (0.4 LPM), an odor stream (0.1 LPM), and a replacement stream (0.1 LPM). For stimulus presentation, a dual synchronous 3-way valve (SV360T041, NResearch, West Caldwell, NJ) was used to switch between the replacement stream and the odor stream that was channeled through odor-containing vials (1:1,000 in mineral oil). The final tubing segment (approximately 25 cm) was fixed on the custom-made imaging chamber and oriented towards the anterior part of the fly’s head.

For Ca^2+^ imaging of all R21B06 neurons, flies (elav^AD^∩R21B06^DBD^ > GCaMP6f, tdTomato^myr^) were prepared as described. Data were acquired as described in previous studies [28] in 20-second sweeps. We used Fiji custom macros to extract responsive cells by subtracting the first frame (baseline) from the peak activity frame. We presented each stimulus 3 times (OCT, MCH) and counted a cell as stimulus specific when 2 or more of the presentations of the same stimulus resulted in neuronal activity. For CAMEL-cell imaging, paired and unpaired conditioned flies (6xCRE^AD^∩R21B06^DBD^ > GCaMP6f, tdTomato^myr^) were alternately prepared and imaged on the same day. Due to technical issues with the olfactory presentation device, 2 complete days were excluded from analysis. We tested each KC manually with 1 odor pulse per odor and then started an automated odor presentation routine consisting of OCT, MCH, benzaldehyde (Merck, Darmstadt, Germany, CAS #100-52-7), and IBA (Merck, Darmstadt, Germany, CAS #110-19-0). We used Fiji [84] and custom recursive batch processing algorithms to enable batch image registration with TurboReg [85] of the whole data set. Images were segmented using a combined approach of Fiji recursive algorithms and manual ROI selection (whole cell body). After segmentation, in each time frame the mean fluorescence intensity of the background was subtracted from the complete image. Custom Matlab (MathWorks, www.mathworks.com) routines were programmed to calculate a normalized trace (f-f_0_/f_0_) such that the baseline is the average mean intensity of the first 28 frames. From the normalized trace, a 5-point running average smoothed trace was generated to extract the peak in each response window. If the peak (within 0–4.5 seconds post stimulus) was 2.33 times greater than the standard deviation of the baseline, it was scored as a response to the stimulus [27]. The same analysis was performed manually. To correct for false positive and false negative responses, in a few cases, response and baseline windows were manually adjusted.

### Statistics

Statistical analysis was performed using GraphPad Prism 7.0 software. Normality of data was tested using D’Agostino Pearson omnibus and Shapiro-Wilk normality tests. Data sets that were significantly different from normal distribution were analyzed using nonparametric tests (Mann-Whitney test and Kruskal-Wallis test) instead of parametric tests (unpaired *t* test and ordinary one-way ANOVA). To correct for multiple comparisons between relevant groups, the Bonferroni (after ANOVA) and Dunn’s (after Kruskal-Wallis) tests were used. More detailed information on statistics and sample sizes are listed in S1 Table for main figures and S2 Table for Supporting Information figures.

## Supporting information

S1 Fig Characterization of the CRE-based reporter tool. (A) Design of the 6xCRE-splitGal4^AD^ vector. (B) Genetic intersection with elav-splitGal4^DBD^ showed that 6 CRE sites resulted in optimal reporter expression in a small and random subset of neurons in the brain. (C) Constitutive labeling of CAMEL neurons in all post-mitotic neurons (elav^DBD^) resulted in labeling of large neuronal populations, including many αβ KCs (LexAop-tdTomato/+; 6xCRE^AD^,elav^DBD^/BrpFRTstopFRT-V5-2A-LexA,UAS-Flp). Scale bars correspond to 50 μm.
(TIF)

S2 Fig CREB2- and LTM-dependent CAMEL activity. (A–B) LTM formation protocol (spaced) resulted in increased CAMEL activity in both brain hemispheres (related to Fig 2F, *n* ≥ 162, 6xCRE^AD^∩R21B06^DBD^ > eGFP). Horizontal lines represent mean ± SEM. Asterisks indicate significant differences between relevant groups (*P < 0.05, ***P < 0.001, Kruskal-Wallis).
(TIF)

S3 Fig Analysis of in vivo responses of CAMEL cells and all R21B06 neurons. (A–B) The number of flies with OCT (CS^+^)-responsive CAMEL cells was significantly higher in the paired compared to the unpaired group 3–4 days after conditioning (A). No difference was observed for MCH (CS^−^) presentation (B) (*n* ≥ 8; 3–10 animals per *n*; total ≥ 63 animals). (C–D) LTM conditioning using MCH as CS^+^ and OCT as CS^−^ resulted in an increase in MCH- but not OCT-responsive animals (*n* ≥ 8; 7–9 animals per *n*; total ≥ 65). (A–D) Each *n* represents data from an independent round of conditioning (6xCRE^AD^∩R21B06^DBD^ > GCaMP6f, tdTomato). (E–G) Analysis of Ca^2+^ signals in all R21B06 neurons in response to OCT and MCH presentation (*n* ≥ 16 animals). Scale bar represents 10 μm. Bars represent mean ± SEM (lines connect data points collected in parallel on the same day). Asterisks indicate significant differences between relevant groups (*P < 0.05, ***P < 0.001, unpaired *t* test [A–C], Mann-Whitney [D]).
(TIF)

S4 Fig Analysis of olfactory acuity and shock responsiveness. (A) All experimental genotypes displayed normal olfactory acuity and shock responsiveness compared to controls (*n* ≥ 7). All data represent mean ± SEM. No significant differences between relevant groups were observed (ANOVA). (B) The experimental genotype showed significantly higher 7-day than 5-day memory performance after correcting the 7-day memory values with a forgetting factor (see Materials and methods). (C–D) Control genotypes showed similar memory performance at days 5 and 7 after correcting for natural forgetting. For box blots: line, median; box, 75th–25th percentiles; whiskers, minimum to maximum (*n* ≥ 8). Asterisks indicate significant differences between relevant groups (*P < 0.05, unpaired *t* test).
(TIF)

S5 Fig Light preference test resolves approach and avoidance behaviors. (A) Optogenetic activation of gustatory receptor neurons involved in bitter tasting (Gr66a) resulted in light avoidance behavior, while activation of the appetitive MBONγ2α’1 (MB077B) elicited light approach behavior (*n* ≥ 10). Bars represent mean ± SEM. Asterisks indicate significant differences between relevant groups (***P < 0.001, ANOVA).
(TIF)

S1 Table Details of statistical analysis for the main figures. (DOCX)

S2 Table Details of statistical analysis for Supporting Information figures. (DOCX)

S3 Table List of primers used in this study. (DOCX)

S1 Data Quantitative observations underlying all the figures. (XLSX)

## Abbreviations

AD: activation domain
ARM: anesthesia-resistant memory
AS: avoidance score
CAMEL: CRE-activity–dependent memory engram label
CRE: cAMP response element
CREB2: cAMP response element binding protein 2
CS: conditioned stimulus
DAN: dopaminergic neuron
DBD: DNA binding domain
EGFP: enhanced GFP
Flp: Flippase
GFP: green fluorescent protein
IBA: Isobutyl-acetate
KC: Kenyon cell
LTM: long-term memory
MB: mushroom body
MBON: MB output neuron
MCH: 4-Methyl-cylohexanol
MTM: mid-term memory
OCT: 3-Octanol
PerI: Performance Index
PI: Preference Index
PN: projection neuron
SEM: standard error of the mean
STM: short-term memory
TNT: tetanus toxin
UAS: upstream activation sequence
