# Supplementary figures and images for: Selective suppression and recall of long-term memories in Drosophila
Source: PLoS Biol. 2019 Aug 27;17(8):e3000400. doi: 10.1371/journal.pbio.3000400 (PMC6711512; doi:10.1371/journal.pbio.3000400)

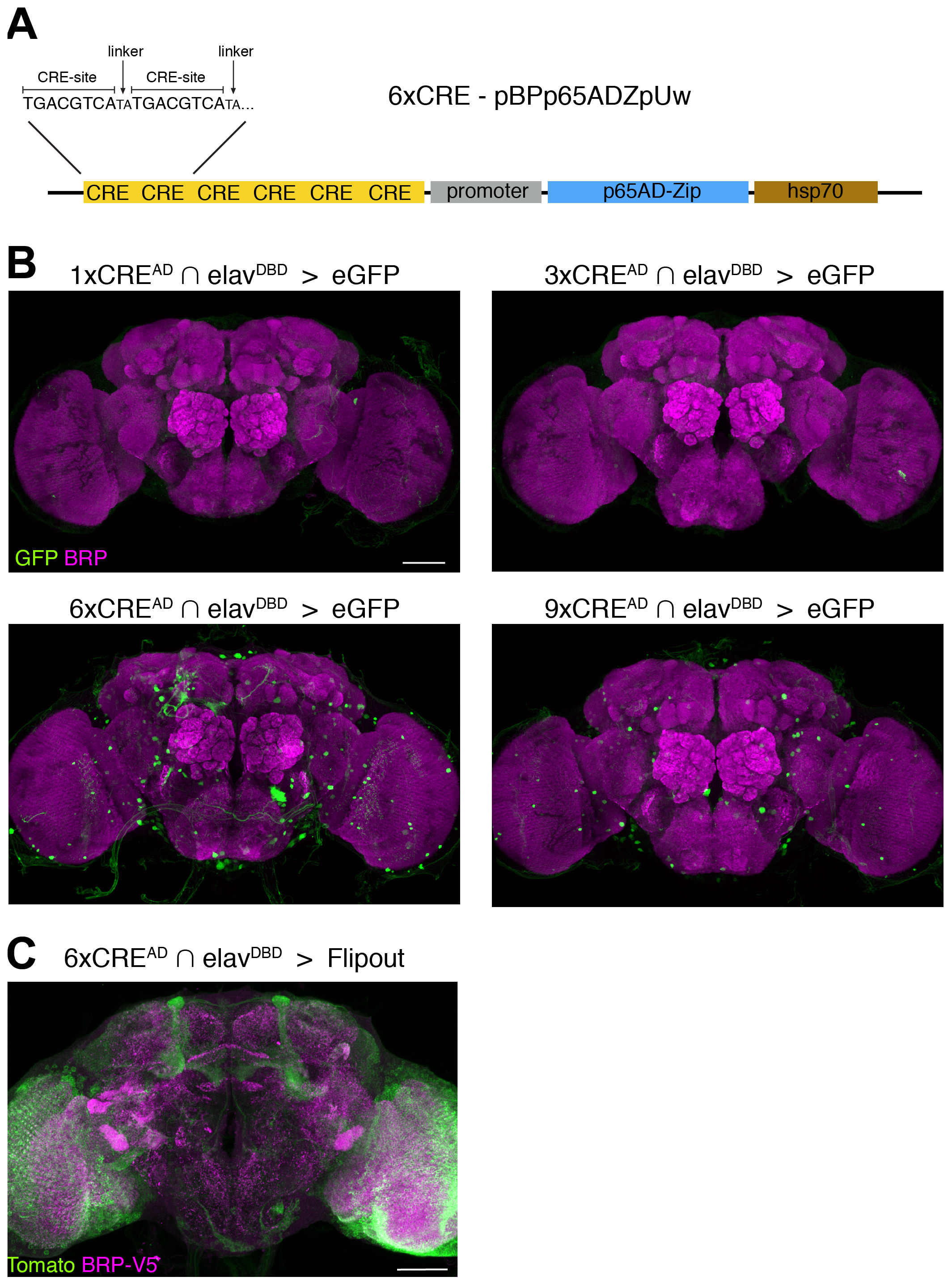

Supplement: S1 Fig — (A) Design of the 6xCRE-splitGal4AD vector. (B) Genetic intersection with elav-splitGal4DBD showed that 6 CRE sites resulted in optimal reporter expression in a small and random subset of neurons in the brain. (C) Constitutive labeling of CAMEL neurons in all post-mitotic neurons (elavDBD) resulted in labeling of large neuronal populations, including many αβ KCs (LexAop-tdTomato/+; 6xCREAD,elavDBD/BrpFRTstopFRT-V5-2A-LexA,UAS-Flp). Scale bars correspond to 50 μm. (TIF) [file pbio.3000400.s001.tif]

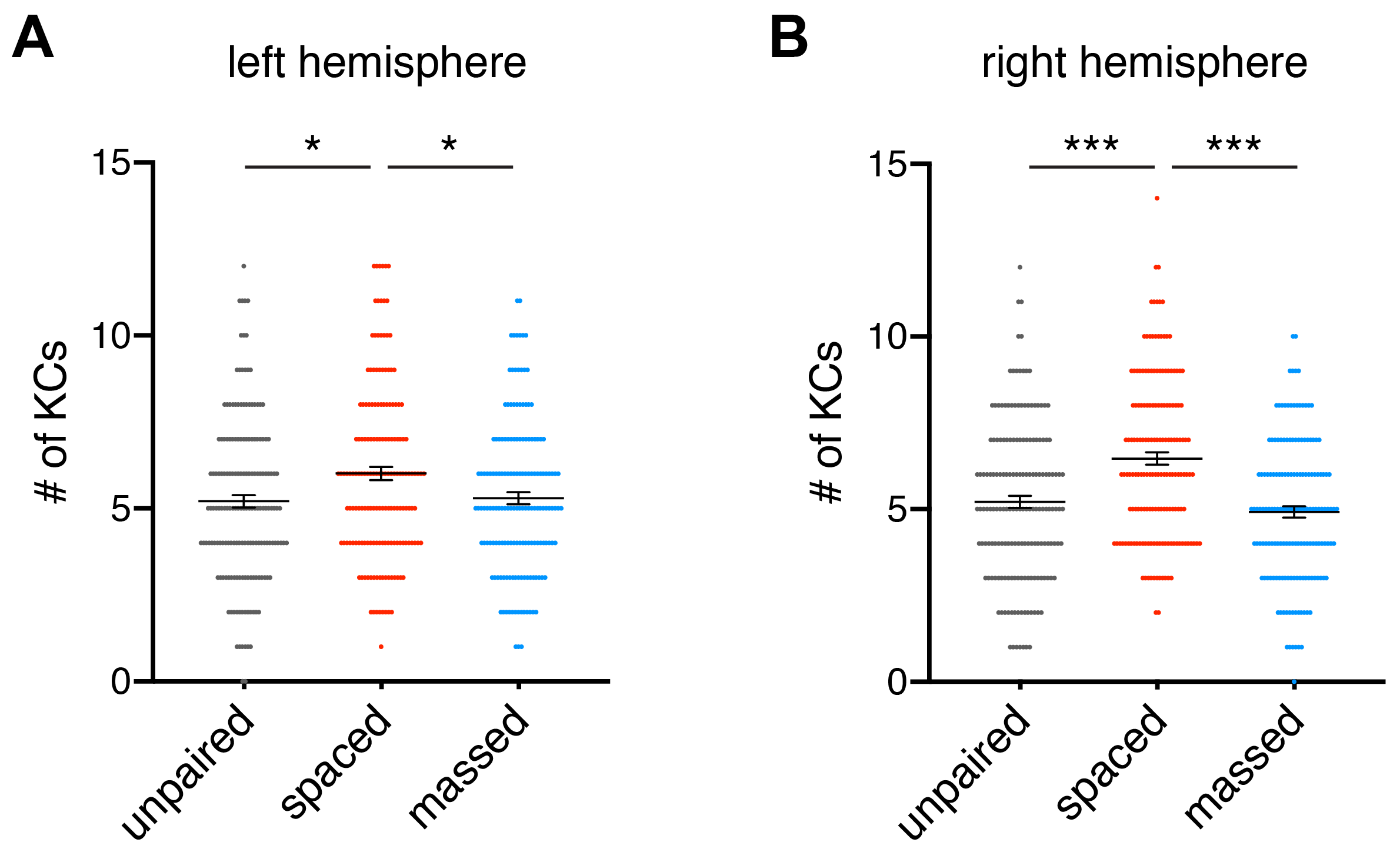

Supplement: S2 Fig — (A–B) LTM formation protocol (spaced) resulted in increased CAMEL activity in both brain hemispheres (related to Fig 2F, n ≥ 162, 6xCREAD∩R21B06DBD > eGFP). Horizontal lines represent mean ± SEM. Asterisks indicate significant differences between relevant groups (*P < 0.05, ***P < 0.001, Kruskal-Wallis). (TIF) [file pbio.3000400.s002.tif]

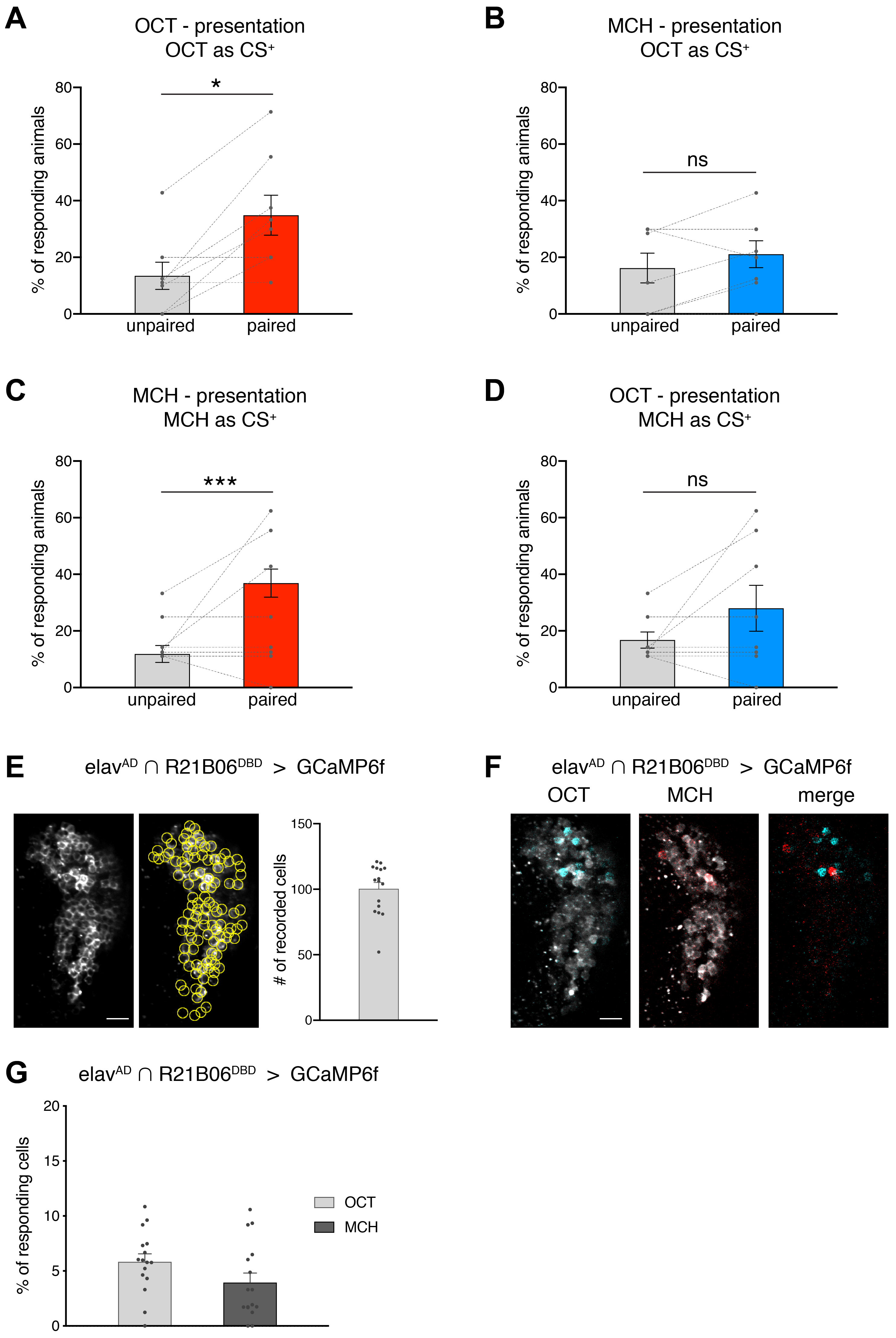

Supplement: S3 Fig — (A–B) The number of flies with OCT (CS+)-responsive CAMEL cells was significantly higher in the paired compared to the unpaired group 3–4 days after conditioning (A). No difference was observed for MCH (CS−) presentation (B) (n ≥ 8; 3–10 animals per n; total ≥ 63 animals). (C–D) LTM conditioning using MCH as CS+ and OCT as CS− resulted in an increase in MCH- but not OCT-responsive animals (n ≥ 8; 7–9 animals per n; total ≥ 65). (A–D) Each n represents data from an independent round of conditioning (6xCREAD∩R21B06DBD > GCaMP6f, tdTomato). (E–G) Analysis of Ca2+ signals in all R21B06 neurons in response to OCT and MCH presentation (n ≥ 16 animals). Scale bar represents 10 μm. Bars represent mean ± SEM (lines connect data points collected in parallel on the same day). Asterisks indicate significant differences between relevant groups (*P < 0.05, ***P < 0.001, unpaired t test [A–C], Mann-Whitney [D]). (TIF) [file pbio.3000400.s003.tif]

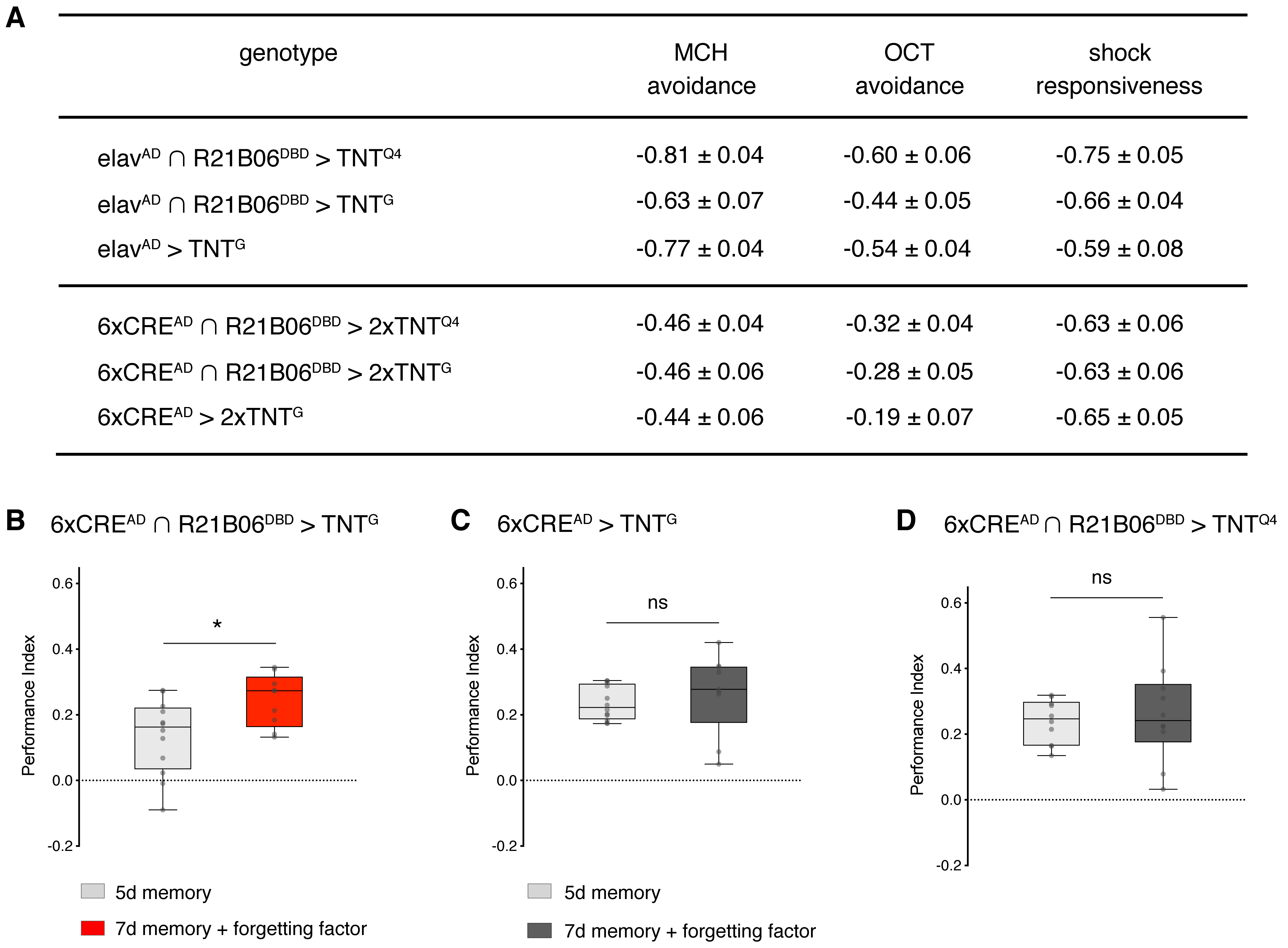

Supplement: S4 Fig — (A) All experimental genotypes displayed normal olfactory acuity and shock responsiveness compared to controls (n ≥ 7). All data represent mean ± SEM. No significant differences between relevant groups were observed (ANOVA). (B) The experimental genotype showed significantly higher 7-day than 5-day memory performance after correcting the 7-day memory values with a forgetting factor (see Materials and methods). (C–D) Control genotypes showed similar memory performance at days 5 and 7 after correcting for natural forgetting. For box blots: line, median; box, 75th–25th percentiles; whiskers, minimum to maximum (n ≥ 8). Asterisks indicate significant differences between relevant groups (*P < 0.05, unpaired t test). (TIF) [file pbio.3000400.s004.tif]

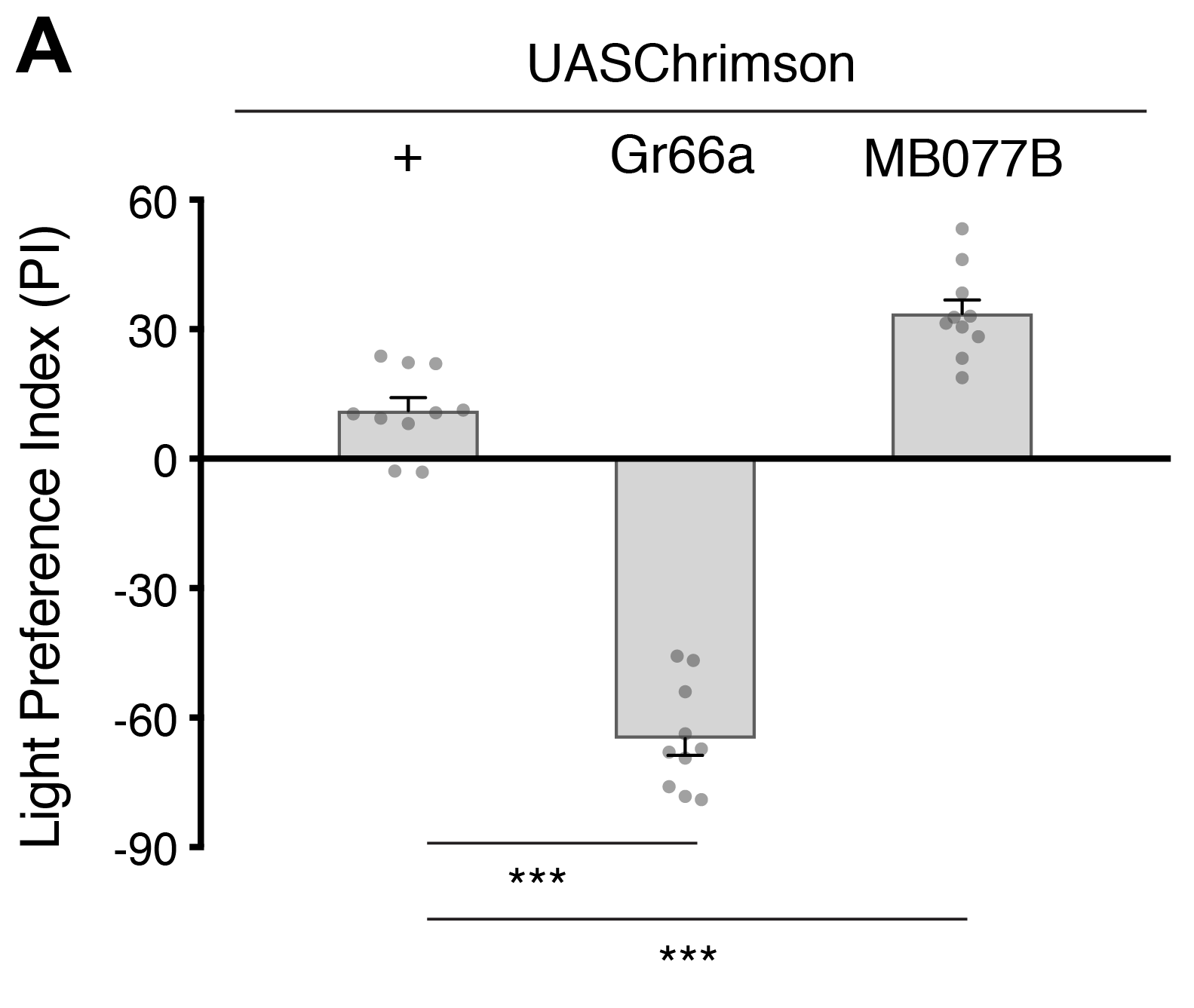

Supplement: S5 Fig — (A) Optogenetic activation of gustatory receptor neurons involved in bitter tasting (Gr66a) resulted in light avoidance behavior, while activation of the appetitive MBONγ2α’1 (MB077B) elicited light approach behavior (n ≥ 10). Bars represent mean ± SEM. Asterisks indicate significant differences between relevant groups (***P < 0.001, ANOVA). (TIF) [file pbio.3000400.s005.tif]
