# Supplementary material for: Selective suppression and recall of long-term memories in Drosophila
Source: PLoS Biol. 2019 Aug 27;17(8):e3000400. doi: 10.1371/journal.pbio.3000400 (PMC6711512; doi:10.1371/journal.pbio.3000400)
Supplement: S1 Table — (DOCX) [file pbio.3000400.s006.docx]

Supplementary Table 1

| **Figure** | **n per group *** | **statistical test** | **F (DFn,DFd) / p values *** | **post hoc test: corrected p values *** |
| --- | --- | --- | --- | --- |
| 1c | 8, 8, 8 | unpaired, one-way ANOVA | 7.759 (2,21) / 0.003 | Bonferroni: 0.0294, 0.0019 |
| 1f | 179, 158, 150 | Kruskal-Wallis test | na / <0.0001 | Dunn’s: <0.0001 (1d vs. 7d), 0.14 (7d vs. 14d), <0.0001 (1d vs. 14d) |
| 1g (1d old) | 13 | unpaired, two-tailed t-test | na / 0.463 | na |
| 1g (7d old) | 10 | Mann-Whitney test, two-tailed | na / 0.867 | na |
| 1g (14d old) | 10 | unpaired, two-tailed t-test | na / <0.0001 | na |
| 1h | 14, 17, 15 | unpaired, one-way ANOVA | 13.17 (2,43) / <0.0001 | Bonferroni: 0.0086, <0.0001 |
| 1i | 172, 173 | Mann-Whitney test, two-tailed | na / 0.0002 | na |
| 2a | 151, 154 | Mann-Whitney test, two-tailed | na / <0.0001 | na |
| 2b | 158, 159 | Mann-Whitney test, two-tailed | na / <0.0001 | na |
| 2d | 172, 168, 162 | Kruskal-Wallis test | na / <0.0001 | Dunn’s: <0.0001, <0.0001 |
| 2e (unpaired) | 10 | unpaired, two-tailed t-test | na / 0.495 | na |
| 2e (spaced) | 10 | unpaired, two-tailed t-test | na / 0.01 | na |
| 2e (massed) | 10 | unpaired, two-tailed t-test | na / 0.388 | na |
| 2f (left hemisphere) | 172, 168, 162 | Kruskal-Wallis test | na / 0.014 | Dunn’s: 0.013, 0.041 |
| 2f (right hemisphere) | 172, 168, 162 | Kruskal-Wallis test | na / <0.0001 | Dunn’s: <0.0001, <0.0001 |
| 3c | 8, 8 | unpaired, two-tailed t-test | na / 0.022 | na |
| 3d | 8, 8 | unpaired, two-tailed t-test | na / 0.961 | na |
| 3e | 8, 8 | unpaired, two-tailed t-test | na / 0.547 | na |
| 3f | 8, 8 | unpaired, two-tailed t-test | na / 0.008 | na |
| 3g | 8, 8 | unpaired, two-tailed t-test | na / 0.417 | na |
| 3h | 8, 8 | unpaired, two-tailed t-test | na / 0.41 | na |
| 4b | 8, 8, 8 | unpaired, one-way ANOVA | 0.25 (2,21) / 0.781 | Bonferroni: >0.9999, >0.9999 |
| 4c | 10, 9, 9 | unpaired, one-way ANOVA | 0.51 (2,25) / 0.607 | Bonferroni: >0.9999, >0.9999 |
| 4d | 15, 16, 13 | unpaired, one-way ANOVA | 4.991 (2,41) / 0.012 | Bonferroni: 0.012, 0.036 |
| 4e | 10, 12, 12 | unpaired, one-way ANOVA | 5.984 (2,31) / 0.006 | Bonferroni: 0.012, 0.011 |
| 4f | 10, 9, 9 | unpaired, one-way ANOVA | 0.084 (2,25) / 0.92 | Bonferroni: >0.9999, >0.9999 |
| 4g | 10, 9, 9 | unpaired, one-way ANOVA | 0.015 (2,25) / 0.985 | Bonferroni: >0.9999, >0.9999 |
| 5b | 18, 18 | unpaired, two-tailed t-test | na / 0.001 | na |
| 5c | 18, 18 | unpaired, two-tailed t-test | na / 0.883 | na |
| 5d | 18, 18 | unpaired, two-tailed t-test | na / 0.007 | na |
| 5d (+retinal) | 18 | two-tailed, one sample t-test | na / 0.0006 | na |
| 5d (-retinal) | 18 | two-tailed, one sample t-test | na / 0.839 | na |
| 5e | 24, 24 | unpaired, two-tailed t-test | na / 0.708 | na |
| 5e (pc1) | 24 | two-tailed, one sample t-test | na / 0.678 | na |
| 5e (pc2) | 24 | two-tailed, one sample t-test | na / 0.753 | na |
| 5f | 18, 18 | unpaired, two-tailed t-test | na / 0.768 | na |
| 5f (+retinal) | 18 | two-tailed, one sample t-test | na / 0.763 | na |
| 5f (-retinal) | 18 | two-tailed, one sample t-test | na / 0.917 | na |
| 5g | 18, 18 | unpaired, two-tailed t-test | na / 0.884 | na |
| 5g (+retinal) | 18 | two-tailed, one sample t-test | na / 0.617 | na |
| 5g (-retinal) | 18 | two-tailed, one sample t-test | na / 0.525 | na |
| 5h (4d) | 18 | two-tailed, one sample t-test | na / <0.0001 | na |
| 5h (5d) | 24 | two-tailed, one sample t-test | na / 0.334 | na |
| 5h (7d) | 18 | two-tailed, one sample t-test | na / 0.285 | na |

* values correspond to graph columns from left to right na = not applicable
