# Supplementary material for: Selective suppression and recall of long-term memories in Drosophila
Source: PLoS Biol. 2019 Aug 27;17(8):e3000400. doi: 10.1371/journal.pbio.3000400 (PMC6711512; doi:10.1371/journal.pbio.3000400)
Supplement: S2 Table — (DOCX) [file pbio.3000400.s007.docx]

Supplementary Table 2

| **Figure** | **n per group *** | **statistical test** | **F (DFn,DFd) / p values *** | **post hoc test *** |
| --- | --- | --- | --- | --- |
| S2a | 172, 168, 162 | Kruskal-Wallis test | na / 0.014 | Dunn’s: 0.013, 0.041 |
| S2b | 172, 168, 162 | Kruskal-Wallis test | na / <0.0001 | Dunn’s: <0.0001, <0.0001 |
| S3a | 8, 8 | unpaired, two-tailed t-test | na / 0.025 | na |
| S3b | 8, 8 | unpaired, two-tailed t-test | na / 0.501 | na |
| S3c | 8, 8 | unpaired, two-tailed t-test | na / 0.0007 | na |
| S3d | 8, 8 | Mann-Whitney test, two-tailed | na / 0.454 | na |
| S4a top (MCH) | 7, 7, 7 | unpaired, one-way ANOVA | 3.22 (2,18) / 0.064 | Bonferroni: 0.052, 0.162 |
| S4a top (OCT) | 7, 7, 7 | unpaired, one-way ANOVA | 2.4 (2,18) / 0.119 | Bonferroni: 0.087, 0.387 |
| S4a top (shock) | 8, 8, 8 | unpaired, one-way ANOVA | 1.63 (2,21) / 0.22 | Bonferroni: 0.657, 0.865 |
| S4a bottom (MCH) | 9, 9, 8 | unpaired, one-way ANOVA | 0.05 (2,23) / 0.956 | Bonferroni: >0.999, >0.999 |
| S4a bottom (OCT) | 9, 9, 9 | unpaired, one-way ANOVA | 1.52 (2,24) / 0.24 | Bonferroni: >0.999, 0.523 |
| S4a bottom (shock) | 8, 7, 8 | unpaired, one-way ANOVA | 0.03 (2,20) / 0.97 | Bonferroni: >0.999, >0.999 |
| S4b | 12, 8 | unpaired, two-tailed t-test | na / 0.023 | na |
| S4c | 12, 9 | unpaired, two-tailed t-test | na / 0.438 | na |
| S4d | 10, 10 | unpaired, two-tailed t-test | na / 0.657 | na |
| S5a | 10, 10, 10 | unpaired, one-way ANOVA | 231.4 (2, 27) / <0.0001 | Bonferroni: <0.0001, 0.0001 |

* values correspond to graph columns from left to right na = not applicable
