# Supplementary material for: Selective suppression and recall of long-term memories in Drosophila
Source: PLoS Biol. 2019 Aug 27;17(8):e3000400. doi: 10.1371/journal.pbio.3000400 (PMC6711512; doi:10.1371/journal.pbio.3000400)
Supplement: S3 Table — (DOCX) [file pbio.3000400.s008.docx]

Supplementary Table 3

| **Primer** | **DNA-Sequence** |
| --- | --- |
| 1xCRE forward | AGCAGGCTCCGCGGCCGCTCTAGATGACGTCATAACTAGTGGCGCGCCGAC |
| 1xCRE reverse | GTCGGCGCGCCACTAGTTATGACGTCATCTAGAGCGGCCGCGGAGCCTGCT |
| 3xCRE forward | AGCAGGCTCCGCGGCCGCTCTAGATGACGTCATATGACGTCATATGACGTCATAACTAGTGGCGCGCCGAC |
| 3x CRE reverse | GTCGGCGCGCCACTAGTTATGACGTCATATGACGTCATATGACGTCATCTAGAGCGGCCGCGGAGCCTGCT |
| 6xCRE forward | GGCTCCGCGGCCGCTCTAGATGACGTCATATGACGTCATATGACGTCATATGACGTCATATGACGTCATATGACGTCAACTAGTGGCGCGCCGAC |
| 6xCRE reverse | GTCGGCGCGCCACTAGTTGACGTCATATGACGTCATATGACGTCATATGACGTCATATGACGTCATATGACGTCATCTAGAGCGGCCGCGGAGCC |
| 9xCRE forward | GGCTCCGCGGCCGCTCTAGATGACGTCATATGACGTCATATGACGTCATATGACGTCATATGACGTCATATGACGTCATATGACGTCATATGACGTCATATGACGTCAACTAGTGGCGCGCCGAC |
| 9x CRE reverse | GTCGGCGCGCCACTAGTTGACGTCATATGACGTCATATGACGTCATATGACGTCATATGACGTCATATGACGTCATATGACGTCATATGACGTCATATGACGTCATCTAGAGCGGCCGCGGAGCC |
| M13  forward | GTAAAACGACGGCCAG |
| M13  reverse | CAGGAAACAGCTATGAC |
| p65AD reverse | GTCCACTGGGGAACACCATCG |
| dest-seq 1  forward | ACTTTCACCAGCGTTTCTGG |
| dest-seq 2  forward | GGCGTATCACGAGGCCCTTTC |

Restriction sites are marked by colour: AscI, XbaI, SpeI, NotI
